# D-Chiro-Inositol Regulates Insulin Signaling in Human Adipocytes

**DOI:** 10.3389/fendo.2021.660815

**Published:** 2021-03-30

**Authors:** Maria Magdalena Montt-Guevara, Michele Finiguerra, Ilaria Marzi, Tiziana Fidecicchi, Amerigo Ferrari, Alessandro D. Genazzani, Tommaso Simoncini

**Affiliations:** ^1^ Molecular and Cellular Gynecological Endocrinology Laboratory (MCGEL), Department of Clinical and Experimental Medicine, University of Pisa, Pisa, Italy; ^2^ Gynecological Endocrinology Center, Department of Obstetrics and Gynecology, University of Modena and Reggio Emilia, Modena, Italy

**Keywords:** Simpson–Golabi–Behmel syndrome cells (SGBS), 17β-estradiol (E2), insulin pathway, insulin receptor substrate (IRS1), D-Chiro-Inositol (D-Chiro-Ins)

## Abstract

D-Chiro-Inositol (D-Chiro-Ins) is a secondary messenger in the insulin signaling pathway. D-Chiro-Ins modulates insulin secretion, the mitochondrial respiratory chain, and glycogen storage. Due to these actions D-Chiro-Ins has been proposed to correct defective insulin function in a variety of conditions characterized by metabolic dysfunction, such as polycystic ovary syndrome (PCOS), obesity, gestational diabetes and fat accumulation at menopause. Since it is unclear whether D-Chiro-Ins directly acts on adipocytes, we aimed to study D-Chiro-Ins’s actions on adipocyte viability, proliferation, differentiation, and insulin-related protein expression using a human adipocyte cell line derived from Simpson–Golabi–Behmel Syndrome (SGBS) which fully differentiates to mature adipocytes. Throughout differentiation, cells were treated with D-Chiro-Ins, 17β-estradiol (E2) or Insulin. Cell viability and proliferation were not affected by D-Chiro-Ins, then D-Chiro-Ins promoted cell differentiation only during the final days of the process, while E2 enhanced it from the first phases. D-Chiro-Ins stimulated lipid storage and the production of big lipid droplets, thus reducing the content of free fatty acids. We also found that D-Chiro-Ins, either alone or in combination with insulin and E2 increased the expression and activation of insulin receptor substrate-1 (IRS1) and glucose transporter type 4 (GLUT4). In conclusion, this work shows that D-Chiro-Ins plays a direct role in the differentiation and in the function of human adipocytes, where it synergizes with insulin and estrogen through the recruitment of signal transduction pathways involved in lipid and glucose storage. These findings give clear insights to better understand the actions of D-Chiro-Ins on fat metabolism in women in physiology and in a variety of diseases.

## Introduction

Adipose tissue contributes to the pathophysiology of a variety of cardiometabolic disorders, such as atherosclerosis, obesity, diabetes mellitus, and the metabolic syndrome (1). Several common conditions in women are influenced by the amount and biological activity of fat tissue, such as menstrual cycle disorders, infertility, polycystic ovary syndrome (PCOS) as well as gestational diabetes (2). The menopausal transition is also characterized by prominent fat tissue changes, with visceral redistribution and increased metabolic perturbance (3).

Adipose tissue has been simplistically considered a storage site for lipids. However, its role as an endocrine organ has recently emerged (4). Adipose tissue is responsible for the synthesis and secretion of several bioactive substances, such as hormones, adipokines, pro-inflammatory cytokines, reactive oxygen species, pro-thrombotic and vasoconstricting factors (5–7). Studies over the years have shown significant differences between men and women in fat distribution and function, suggesting the role of sex hormones. In fact, women experience modifications in fat accumulation, distribution and metabolic activity: all these factors are closely related to hormonal levels’ changes in women, particularly during pregnancy and menopause (8, 9).

Fat estrogen receptors (ERs) α and β modulate lipid and glucose metabolism, regulate total fat mass and contribute to its localization (10, 11). ERα reduces lipogenesis and regulates insulin sensitivity and glucose tolerance, opposing to body weight and white adipose tissue gain (12). In contrast, ERβ appears to be detrimental for the maintenance of glucose and lipid homeostasis (13). Estradiol and insulin receptor share the common signaling chain of the phosphoinositide 3 kinase (PI3K) and serine/threonine kinase (Akt) pathway (14, 15). ERα binds in a ligand-dependent manner to the p85α regulatory subunit of PI3K, thus activating its signaling to Akt. Insulin receptor recruits insulin receptor substrate (IRS) thereby activating PI3K/Akt signaling to glucose transporters (GLUT), ultimately driving a cell’s ability to uptake glucose (16, 17). Therefore, fat cells are the site of as complex regulatory interplay of reproductive and metabolic hormones, and this is relevant to woman’s physiology and disease.

The search of new approaches to address metabolic disorders in women has driven the clinical characterization of pharmacological strategies to enhance insulin function. Inositols are used in clinical practice as dietary supplements due to their role as secondary messengers of insulin receptor (18). Inositols exist under nine stereoisomeric forms, but the most studied are myo-inositol (Myo-Ins) and D-chiro-Inositol (D-Chiro-Ins) (19). D-Chiro-Ins and Myo-Ins are incorporated in the inositol phosphoglycan (IPG) of the cell membrane which is involved in insulin signal transduction. A specific epimerase in mammalian tissues can transform Myo-Ins to D-Chiro-Ins and this epimerization process is compromised during diabetes (20). There is growing evidence suggesting that D-Chiro-Ins deficiency may lead to insulin resistance (IR), diabetes and metabolic disorder as seen in PCOS patients (21–24). D-Chiro-Ins is implicated in the storage of sugars as glycogen, in the regulation of the respiratory chain for the production of ATP, and in the control of insulin secretion by pancreatic β-cells (25). Due to these increasing set of evidence, inositols have been studied in women in the context of a variety of conditions where defective insulin function and excessive body mass index are present, such as PCOS, gestational diabetes and menopausal-related weight gain (26–28).

While it is clear that inositol supplementation is associated with clinical effects on weight and insulin function, the exact role sustained by inositols in glucose metabolism in several insulin-sensitive tissues are less established (29). Namely, whether inositols act directly on human adipocytes is not known. The aim of this paper was to assess the presence of any direct effect of D-Chiro-Ins on human adipocytes, as well as the presence of interactions between D-Chiro-Ins and insulin and estrogen signaling.

## Materials and Methods

### Cell Culture

SGBS cells derived from patients affected by Simpson–Golabi–Behmel syndrome (30, 31). These cells were first isolated by professor Wabitsch in the early 2000s and generously send cell cultures in the proliferative phase (28th generation). They are neither transformed nor immortalized and they provide an almost unlimited source of cells, due to their ability to proliferate for up to 50 generations without losing their characteristics and their ability to differentiate in mature adipocytes.

During the experiments, cells were amplificated not more than until the 35th generation in growth medium. Growth medium was composed by F0 medium [DMEM F-12 (A1443001, Gibco–ThermoFisher), pantothenic acid 17 µM (Sigma-Aldrich), biotin 33 µM (Sigma-Aldrich), L‐glutamine 1% (GlutaMax, 35050061, Gibco–ThermoFisher), and 10,000 U/ml Penicillin-Streptomycin (ATB, 15140122, Gibco–ThermoFisher)] and 10% of fetal bovine serum (FBS, A3160801, Gibco–ThermoFisher). Growth medium was changed every 2 days and cells were cultured at 37°C in a 5% CO2 incubator.

### SGBS Viability and Proliferation

Cell proliferation was measured using trypan blue (T8154 Sigma-Aldrich) and 3-(4,5-dimethylthiazol-2-yl)-5-(3-carboxymethoxyphenyl)-2-(4-sulfophenyl)-2H-tetrazolium kit (MTS kit, ab197010, Abcam). Metabolically active cells can cleave the tetrazolium salt to water soluble formazan. Both assays are suitable for analyzing proliferation, viability, and cytotoxicity.

SGBS cells were plated in 6-wells plate for trypan blue assay (60,000 cells/well) or in a 96-well plate for MTS assay (4,000 cells/well) then, were exposed to increase concentration of D-Chiro Inositol (D-Chiro-Ins, Farmitalia, Italy) and 17β-estradiol (E2, E2758 Sigma-Aldrich) for 72 h. Before each cell proliferation experiment, medium was replaced over night with medium containing 10% charcoal stripped-fetal bovine serum (CS-FBS, 12676029, Gibco–ThermoFisher). Control cells were treated with ethanol 0.01% final concentration, since it is the solvent for E2. Every 24 h fresh mediums and treatments were replaced. Control and treatment were performed in triplicate.

When cell viability-proliferation was evaluated by trypan blue assay, SGBS cells treated were washed with PBS, trypsinized and collected then, cell suspensions were mixed with an equal volume of 0.4% (w/v) trypan blue solution to count the live (clear) and dead (blue) cells in a hemocytometer. Cell viability was calculated by means of the formula: viability (%) = (viable treated cells/total control cells) × (viable treated cells/viable control cells) × 100.

While for MTS assay, every 24 h of treatments, the solution reagent is simply added into the cell culture media without further steps. The amount of formazan that is produced was analyzed 3 h after its addition by the measurement of absorbance at wavelength 490 nm (Chromate 4300, Awareness technology). Cell proliferation was displayed as a relative quantity to control cells.

### Adipocyte Differentiation Process

Differentiation was started when SGBS cells reached confluency (day 0) in serum-containing medium. Preadipocytes were washed with PBS, and then changed to the primary differentiation medium called Quick-Diff, which contains: 3FC medium [F0 medium, 0.01 mg/ml transferrin, 20 nM insulin, 20 nM cortisol, and 0.2 nM Triiodothyronine (T_3_)] with 25 µM dexamethasone, 250 mM 3-Isobutyl-1-methylxanthine (IBMX) and 10 mM Rosiglitazone, all from Sigma-Aldrich. At day 4, medium was changed and replaced with 3FC medium with no previous washing with PBS. 3FC medium was changed every 4 days until the end of the differentiation period. Cells were considered fully mature 28 days post-differentiation when clearly visible lipid droplets were present (32–34).

### Adipogenic Differentiation Rate—Oil Red O Staining

Adipogenic differentiation rate was determined *via* oil red O staining at time points 0, 4, 10, 16, 22- and 28-day treatment. Since the first day and during the whole differentiation period, cells were treated with 10^−9^ M E2, 10^−8^ M D-Chiro-Ins, 10^−7^ M Insulin and 0.01% ethanol (E2 vehicle). Medium and treatments were changed as scheduled in the differentiation protocol (every 4 days). SGBS cells were grown in 6-well plates (60,000 cells/well) containing sterile 18 mm glass cover slips in growth medium. Medium was removed at the above time points, then cells were rinsed with PBS and fixed for 30 min with 4% paraformaldehyde at room temperature; subsequently, fixed cells were rinsed twice with PBS. Afterwards, the cells were stained with a working solution of Oil-Red O (O1391, Merck-Sigma-Aldrich) for 30 min in agitation (35). The working solution was obtained by diluting the stock solution 2:3 with distilled water, yielding a concentration of 0.2% oil red O in 40% isopropanol; it was prepared freshly solution for all experiments and filtered once immediately before use. In addition, dapi staining was used to determine the number of nuclei and gross cell morphology, helping to make a rate relation between the number of cells and the red intensity of the lipids-droplets.

Cells were visualized with an immunofluorescence microscope (Olympus BX41) and digitalized with a digital camera (Olympus DP70). For each treatment, 8 photographs of different areas were taken and lipid droplets were then analyzed in terms of quantity and dimension using NIH ImageJ software (36).

### Protein Expression in Differentiated SGBS Cells—Western Blot Assays

SGBS cells were plated on 100 mm tissue culture and differentiated as previously described. Differentiated adipocytes from 28 days, were treated with 10^−9^ M E2, or 10^−8^ M D-Chiro-Ins, or 10^−7^ M insulin and combinations for 24 h in a low-glucose medium (5.5 mM glucose, DMEM 11054020 ThermoFisher). Preliminary experiments were performed, showing that the 24-hour time point was optimal to study our primary objective (maximum pIRS1/IRS1 ratio expression with D-Chiro-Ins). Prior to treatments, for one-hour, cells were deprived of glucose by incubating them with DMEM medium without glucose (11054020, ThermoFisher). After treatments, cells were collected on ice with lysis buffer that contained 50 mM Tris–HCl—pH 7.4, 1 mM EDTA, 1% IGEPAL, proteases inhibitor cocktail PIC (P2714, Sigma-Aldrich), and phosphatase inhibitor cocktail PHIC-3 (P0044, Sigma-Aldrich). Protein concentration was determined using BCA assay (23235, Pierce Micro BCA Protein Assay Kit, ThermoFisher). Samples containing 40 μg of protein were separated on TGX Stain-Free Fast Cast 10% Acrylamide SDS kit gels (161-0183 Bio-Rad) and transferred by Trans-Blot Turbo Transfer System to a 0.2 μm PVDF membrane (1704157, Bio-Rad). Antibodies against Glucose Transporter 4 (GLUT4, MA5-17176, ThermoFisher), phospho-GLUT4 (Ser488) (p-GLUT4, ab188317, Abcam), Insulin Receptor Substrate-1 (IRS1, sc-559, Santa Cruz Biotechnology), phospho-IRS1 (Tyr941) (p-IRS1, sc-17199-R, Santa Cruz Biotechnology), Serine/Threonine-Protein Kinases (AKT1, sc-1618, Santa Cruz Biotechnology), Phospho-AKT1 (Ser473) (p-AKT, 44-621G, Thermo Scientific) and GAPDH (sc-47724, Santa Cruz Biotechnology) were used. Primary and secondary antibodies were incubated with standard technique. Immunodetection was visualized by chemiluminescence and digitalized with Quantity One software (BioRad). Optical densitometry (OD) analysis of the bands was performed using the NIH ImageJ software. OD values were expressed as the ratio of each band versus their respective loading control GAPDH.

### Statistical Analysis

GraphPad Prism 7 software (GraphPad Prism) was used for the statistical analysis. When evaluating growth curve data (proliferation slope) linear regression analysis was performed. The normality of the variables was verified with Shapiro–Wilk. In accordance, ordinary or repeated measures (RM) one-way ANOVA, followed by Dunnett’s multiple comparisons test was performed when comparing control group against treatments. For assessing if treatments had the same effect at every single time point, two-way RM matched ANOVA was performed followed by Dunnett’s multiple comparisons test between experimental treatments and the control group. A value of p <0.05 was considered statistically significant. All data are presented as mean ± standard error of the mean (SEM).

## Results

### D-Chiro-Ins Does Not Affect Adipocyte Viability and Proliferation Rate

**Figure 1:**
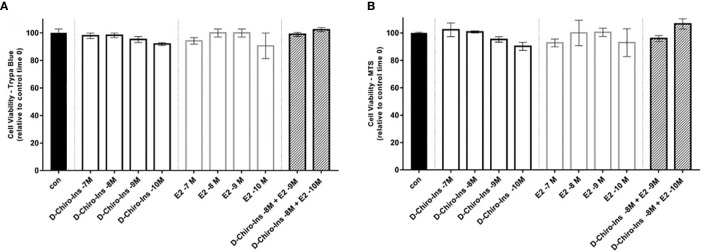
D-Chiro-Ins does not affect SGBS cells viability. SGBS cells viability was measured by trypan blue assay **(A)**, or MTT assay **(B)** after 24-hour treatment with increasing concentration of D-Chiro-inositol (D-Chiro-Ins), 17β-estradiol (E2) or combinations of D-Chiro-Ins and E2. All the results represent the media out of three independent experiments, each of them performed in triplicates. Data are expressed as mean ± SEM. One-way ANOVA was performed followed by Dunnett’s multiple comparisons. No significative difference was found between treatments and control after 24-hour treatment.

**Figure 2:**
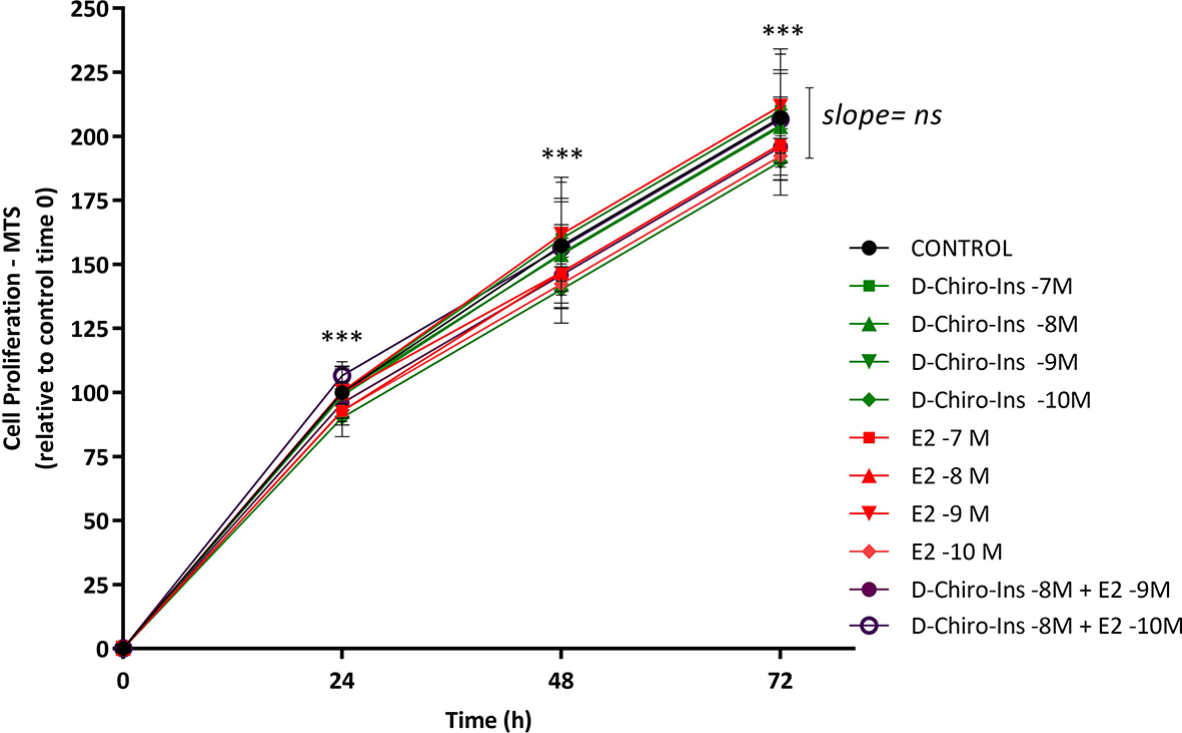
D-Chiro-Ins does not affect SGBS cells proliferation rate. SGBS cells proliferation was measured by MTT assay for 72-hour treatment with increasing concentration of D-chiro-Inositol (D-Chiro-Ins), 17β-estradiol (E2) or their combinations. All the results are expressed as mean ± SEM, obtained after three independent experiments, each of them performed in triplicates. To analyse the proliferation slope, linear regression analysis was performed; however, the difference between the slopes was not significant (ns: non-significant). In addition, to compare each treatment in the time-response curve, a two-way RM ANOVA was performed followed by Dunnett’s multiple comparisons test; treatments did not affect the cell proliferation rate, however, only time progression was found to affect cell proliferation in a significative way (***p < 0.001 versus every preceding time).

To characterize D-Chiro-Ins actions on pre-adipocytes, we first studied whether it could affect cell viability and proliferation with Trypan Blue and MTS assays. SGBS steroid-deprived cells were treated for 72 h with increasing concentration of D-Chiro-Ins (10^−7^ to 10^−10^ M), E2 (10^−7^ to 10^−10^ M), and their combinations: D-Chiro-Ins (10^−8^ M) plus E2 (10^−9^ and 10^−10^ M). Under standard conditions, pre-adipocytes had a doubling time of 38 ± 1 h. Neither D-Chiro-Ins nor E2 affected viability and proliferation of pre-adipocytes (**Figures 1A, B** and **2**).

### Cell Differentiation of Human SGBS Preadipocytes Into Mature White and Brown Adipocytes

**Figure 3:**
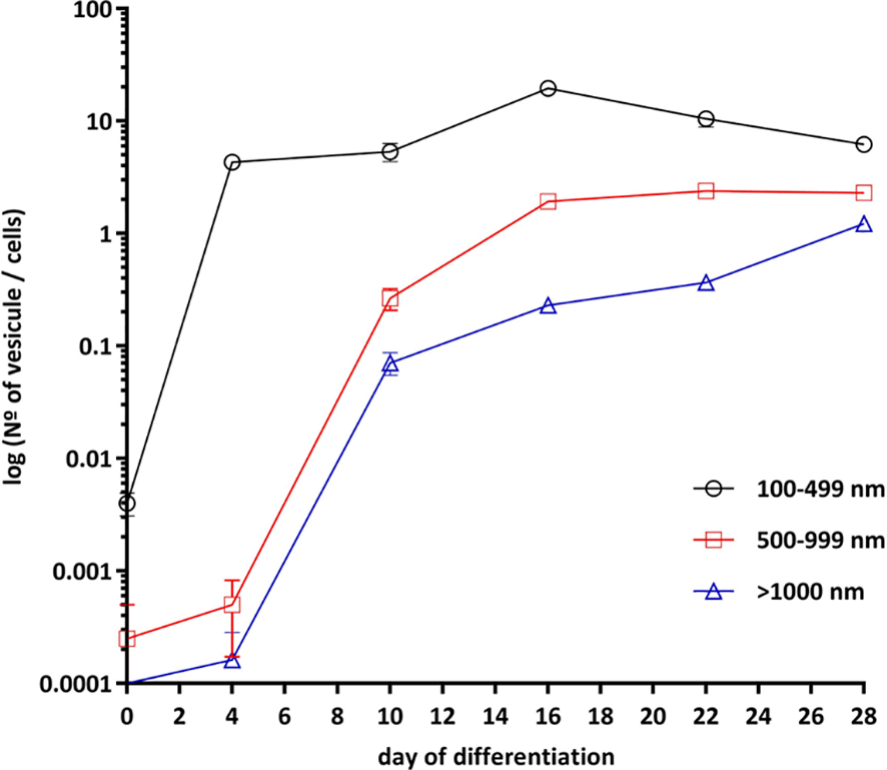
Adipogenic differentiation of SGBS cells. SGBS cells differentiation was according to differentiation protocol. Cells imaging was taken at days 0, 4, 10, 16, 22 and 28 of differentiation. The presence of lipid droplets was assessed at magnification of 20×. Analysis of the different size of lipid droplets (100–499, 500–999 and >1000 nm) was perform with by ImageJ. All data are presented as mean ± SEM after three independent experiments.

Cell differentiation was studied by measuring changes in lipid droplets as shown in **Figure 3**. Before differentiation, SGBS cells appeared elongated, spindled, with a little nucleus and no lipid droplets. They were also distant from each other and arranged according to a casual pattern. At day 4, cells changed to a rounder shape, lipid droplets started to be accumulated, and cellular elements started to overlap due to overcrowding. At day 10, lipid droplets were clearly visible. At day 16, 90% of cells reached a good level of differentiation with small, well-formed and numerous lipid droplets; at this time point, differentiated SGBS cells appeared to be like brown/beige adipocyte phenotype of adipose tissue cells (32–34). At day 22, lipid droplets became less numerous and larger. At day 28, cells displayed a fully differentiated mature white adipocyte phenotype and lipid droplets were found to be bigger in size, while less numerous (32–34) (**Figure 3**).

### D-Chiro-Ins Modulates Adipocyte Differentiation

**Figure 4:**
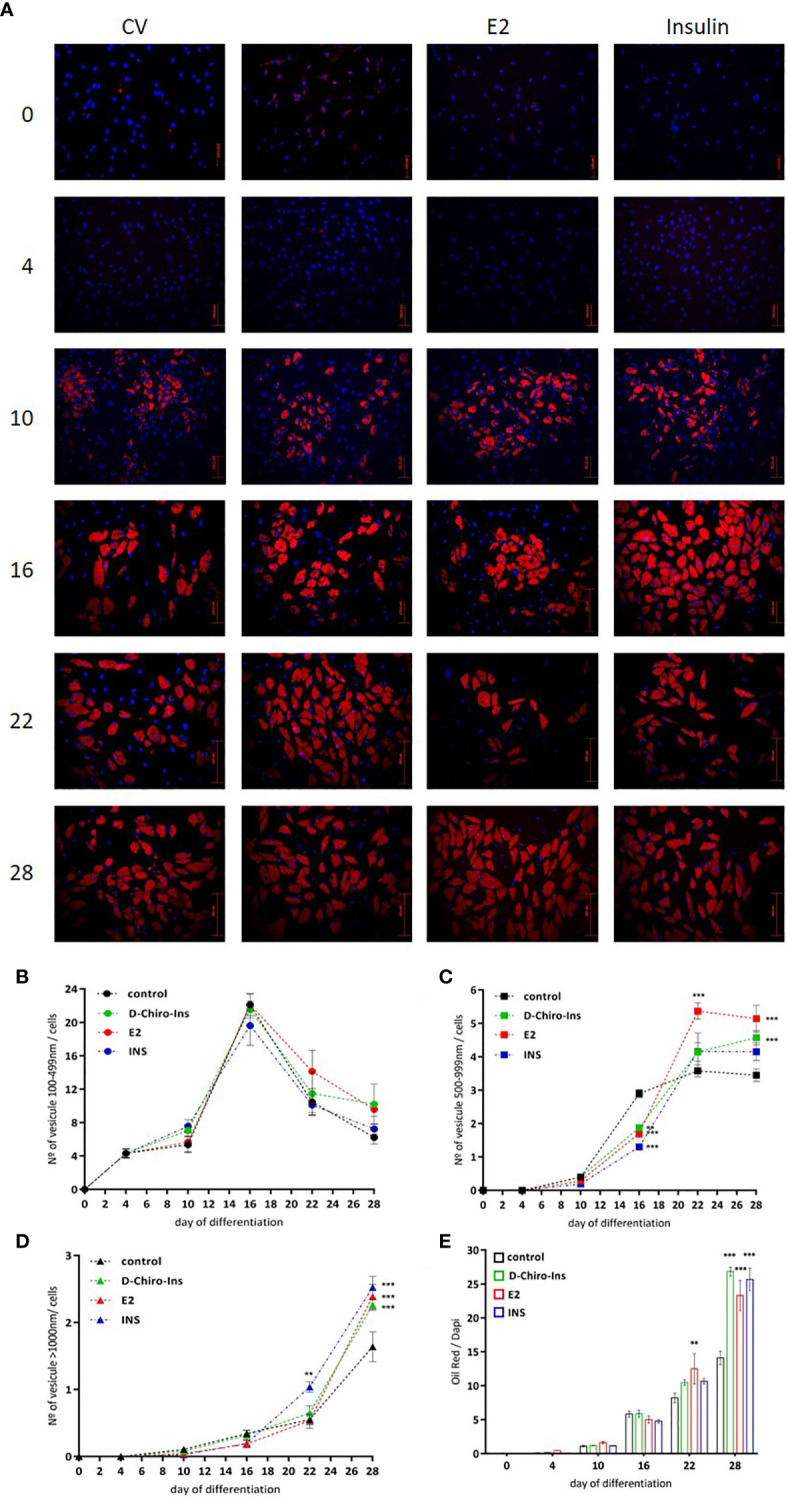
D-Chiro-Ins modulates SGBS cells differentiation. **(A)** SGBS were treated with D-Chiro-Ins 10^−8^ M, E2 10^−9^ M, INS 10^−7^ M or without treatment (cv, control) during differentiation according to differentiation protocol. Lipid droplets (LD) formation rate was observed with oil red/dapi staining, on days 0, 4, 10, 16, 22 and 28 of the differentiation process. Images representative of three independent experiments taken with immunofluorescence microscope assessed at magnification of 20×. Red: oil red; blue: dapi; bar: 200 nm. **(B–D)** Analysis of different size of LD produced during the differentiation: 100–499, 500–999 and >1000 nm size. **(E)** Analysis of lipid accumulation during differentiation, the results were expressed as ratio of the oil red intensity of lipids-droplets and dapi as a nuclear staining dye (oil red/dapi). All the results are presented as mean ± SEM after three independent experiments. Results were analyzed statistically by two-way RM ANOVA. Differences between experimental treatments and control group at the same matching time were analyzed by Dunnett’s multiple comparisons test (**p <0.01, ***p <0.001 versus control at the corresponding time). D-Chiro-Ins, D-chiro-Inositol; E2, 17β-estradiol, INS, insulin.

With the purpose of characterizing whether D-Chiro-Ins could affect adipocyte differentiation, SGBS cells were treated with D-Chiro-Ins 10^−8^ M, E_2_ 10^−9^ M or insulin 10^−7^ M during the differentiation process. Lipid droplets formation rate was observed at 0, 4, 10, 16, 22 and 28 days. Lipid accumulation was estimated in living cells by phase contrast microscopy and confirmed with oil red O assay staining under fluorescence microscopy (**Figure 4A**). During adipocyte differentiation small, medium and large lipid droplets rate formation showed diverse outcomes. Small lipid droplets (100–499 nm) started to be more visible on day 10. At day 16, small lipid droplets showed the highest expression and decreased thereafter. The formation of small lipid droplets progressively decreased until full differentiation. None of the treatments affected the formation of the small lipid droplets (**Figure 4B**).

Medium-size lipid droplets (500–999 nm) became visible from day 16; in addition, cells treated in presence of D-Chiro-Ins, E2 and insulin showed significantly lower numbers of medium droplets compared to control cells (E2 and insulin p <0.001; D-Chiro-Ins p = 0.004). At day 22, medium-size lipid droplets reached the highest amount; in particular, cells treated with E2 showed a significantly higher number of medium droplets compared to controls (p <0.001). When full differentiation was reached, at day 28, the cells differentiated in presence of D-Chiro-Ins or E2 had the highest number of medium-size lipid droplets (both p <0.001) (**Figure 4C**).

Similar results were found with large-size (>1,000 nm) lipid droplets formation. Large lipid droplets became visible from day 22, and cells treated with insulin showed a higher number of large droplets compared to controls (p = 0.001). At day 28, cells treated with D-Chiro-Ins, E_2_ or insulin all displayed significantly higher formation of large droplets compared to controls (all p <0.001) (**Figure 4D**).

In parallel, lipid accumulation was also assessed as the ratio between oil red intensity (which measures lipids) and DAPI intensity (which highlights the nucleus). An interaction between exposure to treatments and time was identified (interaction and treatment: p <0.001; time p = 0.009). At day 16, cells differentiated in presence of D-Chiro-Ins, E2 or insulin did not show differences compared to controls. However, at day 22, cells treated with E2 displayed a higher lipid accumulation rate (p = 0.007) and, at day 28, cells treated with D-Chiro-Ins, E2, or insulin all presented a higher lipid accumulation compared to controls (all p <0.001) (**Figure 4E**).

### D-Chiro-Ins Modulates the Expression of IRS1 in Mature Adipocytes

**Figure 5:**
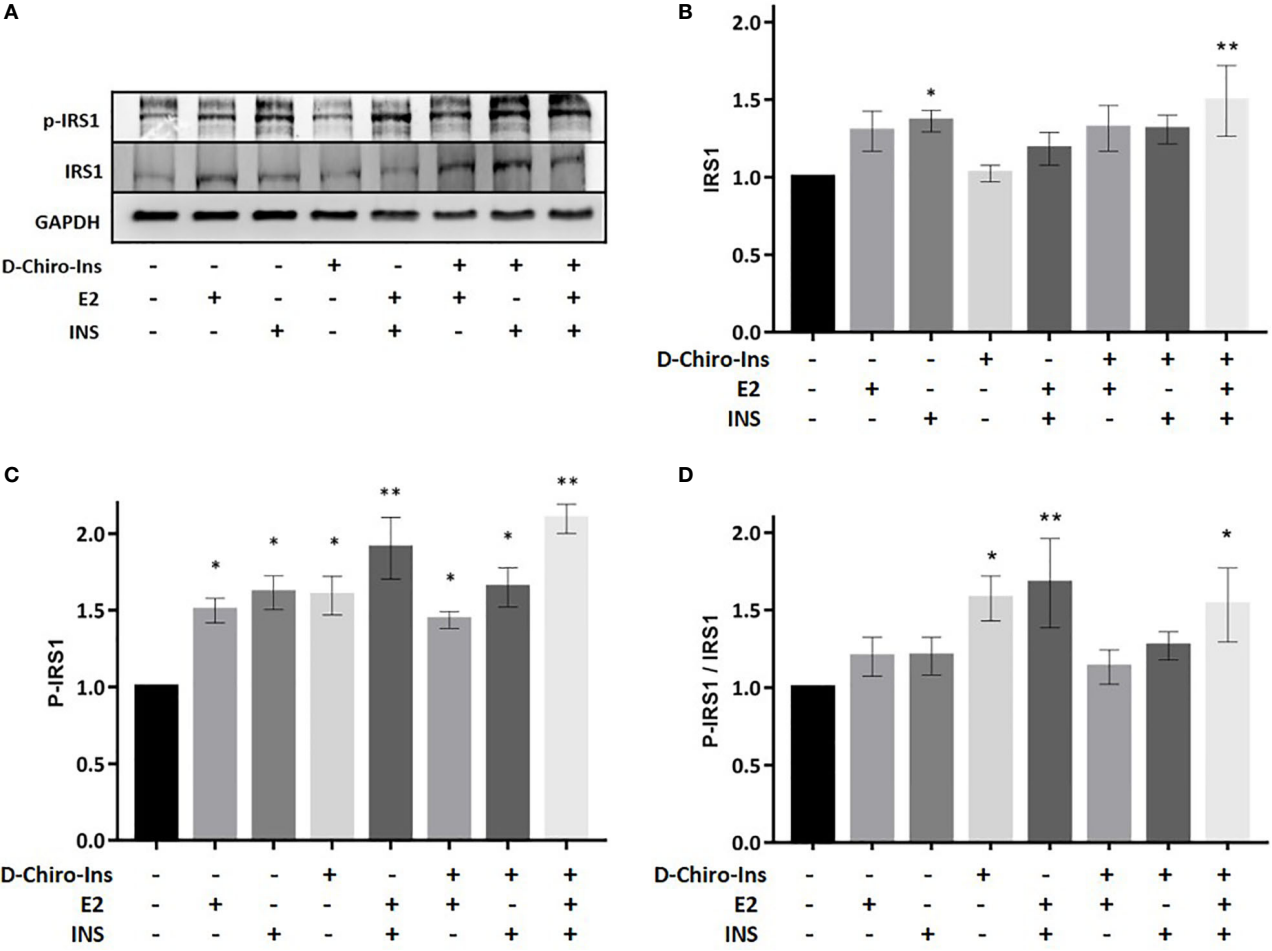
D-Chiro-Ins modulates the expression and phosphorylation of IRS1 in mature adipocyte. Mature SGBS adipocytes cells were treated with D-Chiro-Ins 10^−8^ M, E2 10^−9^ M or INS 10^−7^ M, and combinations for 24 ho. Cell lysates were immunoblotted with an anti-IRS1 antibody, anti-p-IRS1 antibody, and anti-GAPDH antibody. **(A)** Panels show representative blot of IRS1, p-IRS1^Tyr941^ and GAPDH. The expression level of IRS1 and p-IRS1 was normalized with the protein amount of GAPDH, relative to the control. In the p-IRS1 Tyr^941^ blot the band subjected to quantification is the lower, corresponding to 170 kDa. **(B–D)** Analysis of the relative expression level of IRS1, p-IRS1 and the ratio p-IRS1/IRS1. Values are presented as mean ± SEM of three independent experiments. Results were analyzed statistically by one-way RM ANOVA followed by Dunnett’s multiple comparisons test (*p < 0.05, **p < 0.01 versus control). D-Chiro-Ins, D-chiro-Inositol; E2, 17β-estradiol, INS, insulin; IRS1, insulin receptor substrate 1; p-IRS1, phospho-IRS1; GAPDH, glyceraldehyde-3-phosphate dehydrogenase.

**Figure 6:**
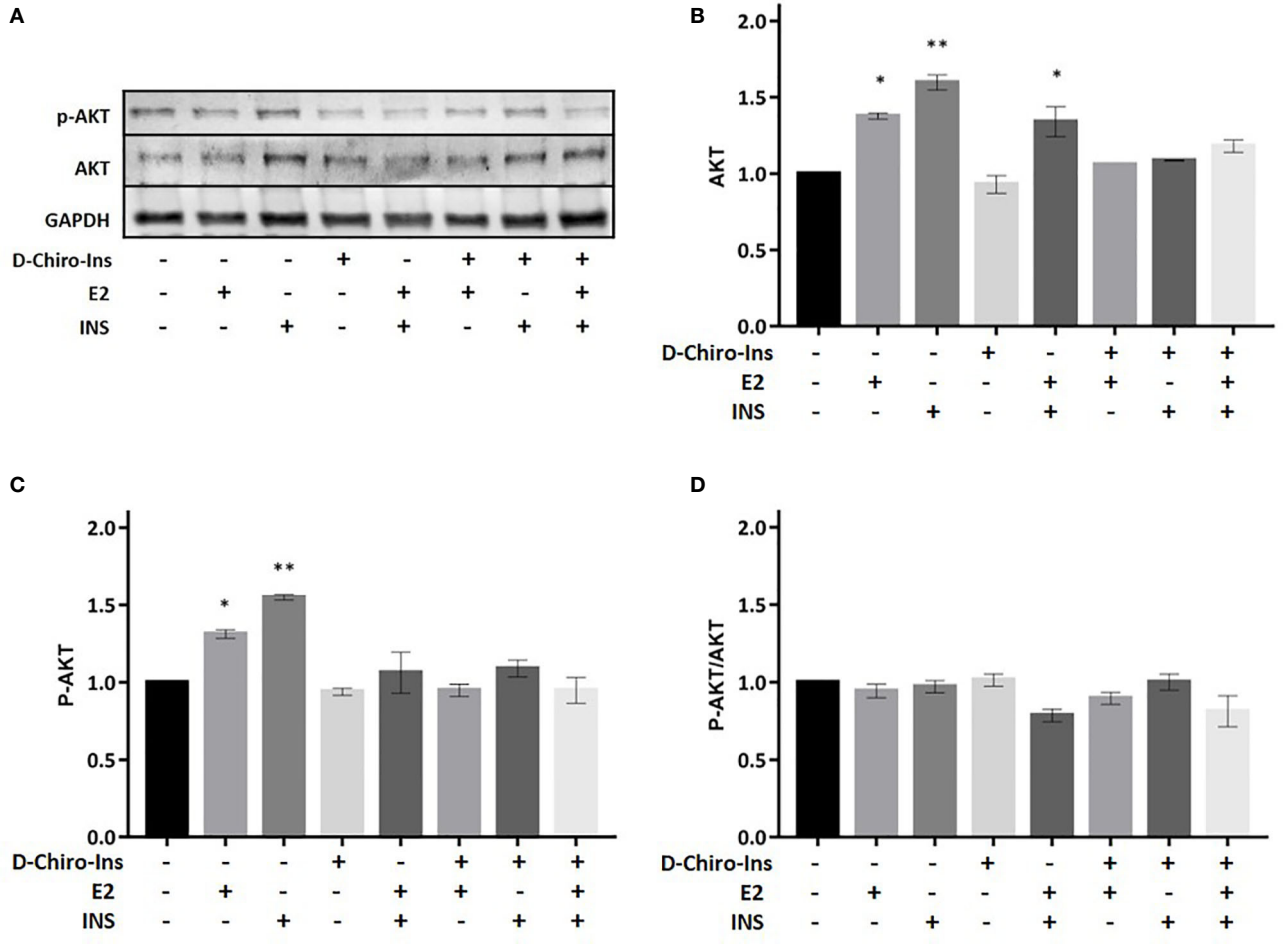
D-Chiro-Ins does not modulate the expression of Akt. Mature SGBS adipocytes cells were treated with D-Chiro-Ins 10^−8^ M, E2 10^−9^ M or INS 10^−7^ M, and combinations for 24 h. Cell lysates were immunoblotted with an anti-Akt antibody, anti-p-Akt antibody, and anti-GAPDH antibody. **(A)** Panels show representative blot of Akt, p-Akt^Ser473^ and GAPDH. **(B–D)** Analysis of the relative expression level of Akt, p-Akt and the ratio p-Akt/Akt. The expression level of Akt and p-Akt was normalized with the protein amount of GAPDH, relative to the control. Values are presented as mean ± SEM of three independent experiments. Results were analyzed statistically by one-way RM ANOVA followed by Dunnett’s multiple comparisons test (*p < 0.05, **p < 0.01 versus control). D-Chiro-Ins, D-chiro-Inositol; E2, 17β-estradiol, INS, insulin; GAPDH, glyceraldehyde-3-phosphate dehydrogenase.

**Figure 7:**
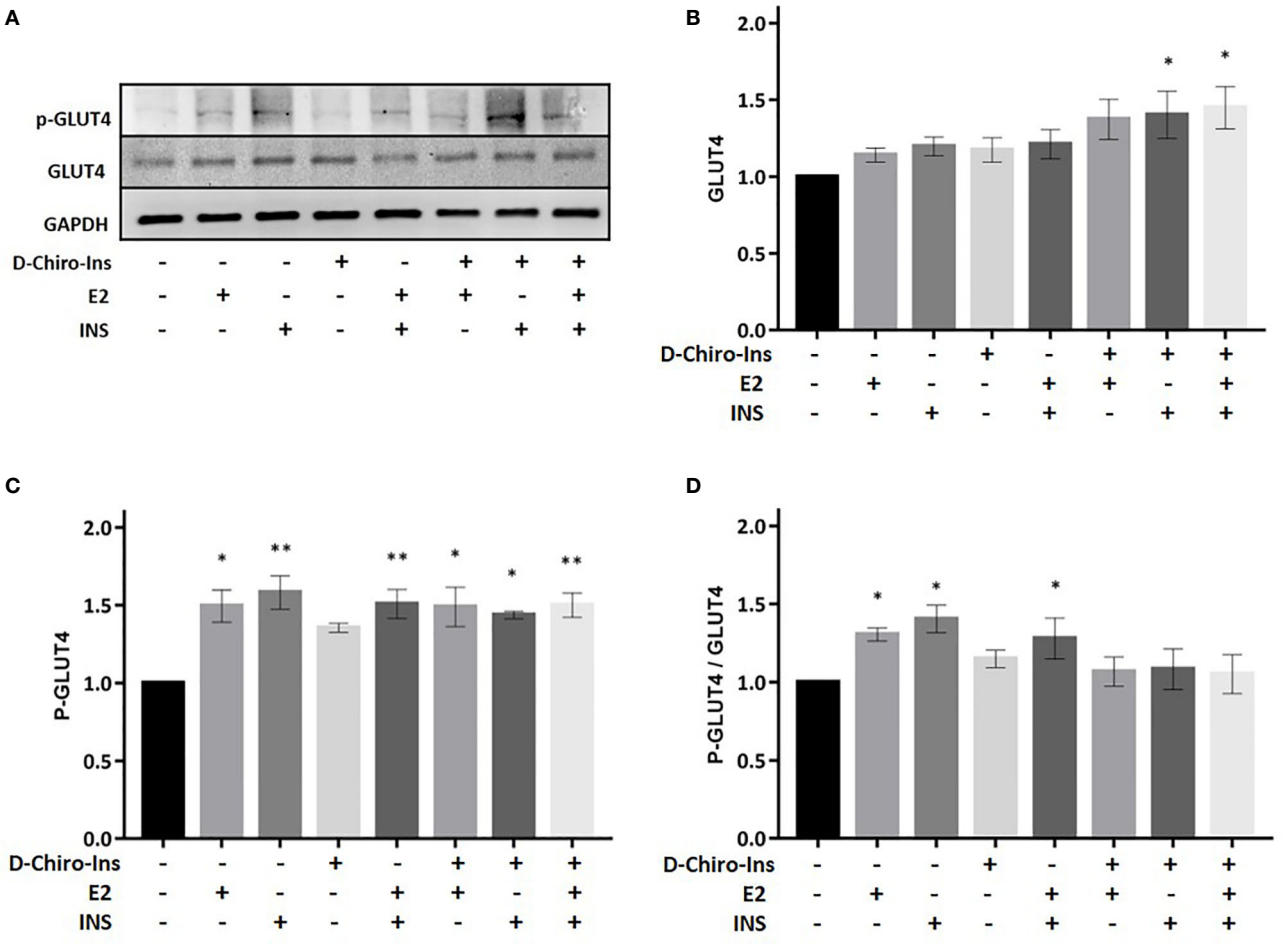
D-Chiro-Ins does not modulate the expression of GLUT4. Mature SGBS adipocytes cells were treated with D-Chiro-Ins 10^−8^ M, E2 10^−9^ M or INS 10^−7^ M, and combinations for 24 h. Cell lysates were immunoblotted with an anti-GLUT4 antibody, anti-p-GLUT4 antibody, and anti-GAPDH antibody. **(A)** Panels show representative blot of GLUT4, p-GLUT4 ^S488^ and GAPDH. **(B–D)** Analysis of the relative expression level of GLUT4, p-GLUT4 and the ratio p-GLUT4/GLUT4; expression that was normalized with GAPDH and relative to the control. Values are presented as mean ± SEM of three independent experiments. Results were analyzed statistically by one-way RM ANOVA followed by Dunnett’s multiple comparisons test (*p < 0.05, **p < 0.001 versus control). D-Chiro-Ins, D-chiro-Inositol; E2, 17β-estradiol, INS, insulin; GLUT4, Glucose Transporter 4; p-GLUT4, phospho-GLUT4; GAPDH, glyceraldehyde-3-phosphate dehydrogenase.

We then assessed whether D-Chiro-Ins could modulate insulin signaling in adipocytes by altering the expression or activity of relevant signaling intermediates. Fully differentiated adipocytes were treated with D-Chiro-Ins 10^−8^ M, E2 10^−9^ M or INS 10^−7^ M, or with combinations for 24 h in low-glucose medium. Western Blot analysis was then performed to assess the absolute expression and phosphorylation status of IRS1, Akt and GLUT4 (**Figures 5**–**7**).

Insulin receptor substrate-1 (IRS1) is phosphorylated by insulin receptor and functions as an adaptor protein for the activation of Akt signaling (37). Treatment with insulin alone or in combination with D-Chiro-Ins + E2 (D-Chiro-Ins + E2 + INS) increased the expression of IRS1 in differentiated SGBS adipocytes (p = 0.045; p = 0.004, respectively) (**Figure 5B**). In addition, all treatments induced the phosphorylation of IRS1 (E2: p = 0.01; INS: p = 0.02; D-Chiro-Ins: 0.03; E2 + INS: p = 0.04; D-Chiro-Ins + E2: p = 0.005; D-Chiro-Ins + INS: p = 0.03; D-Chiro-Ins + E2 + INS: p = 0.001) (**Figure 5C**), which signifies activation of the protein. Insulin and estradiol alone do not increase the protein ratio due to the parallel increase in both the native and phosphorylated form; however, their combination promotes activation of pIRS1/IRS1 ratio. D-Chiro-Ins alone or with insulin and/or estradiol turned into an increased pIRS1/IRS1 ratio (**Figure 5D**), suggesting that D-Chiro-Ins modulates the signal transduction pathway of insulin receptor in adipocytes.

Akt is involved in various biological processes, including adipocyte differentiation, glycogen synthesis and glucose uptake (38). We studied if Akt expression or phosphorylation on serine 473 could be altered by D-Chiro-Ins. D-Chiro-Ins did not increase the expression of Akt nor its phosphorylation/activation (**Figure 6**). This would indirectly suggest that D-chiro-Ins effect may not selectively involve Akt pathway in the studied condition. Still, treatment with E2 or insulin or the combination induced Akt expression and activation (E2: p = 0.01, p = 0.02; INS: p = 0.001, E2 + insulin: p = 0.003) (**Figures 6B, C**). However, due to a parallel increase in both the native and phosphorylated form p-Akt/Akt ratio was not observed (**Figure 6D**).

GLUT4 is an insulin responsive glucose transporter involved in the several conditions, including type 2 diabetes (39). D-Chiro-Ins alone did not induce the expression of GLUT4 nor its phosphorylation (**Figure 7**). However, treatment with the combination of D-Chiro-Ins + insulin or D-Chiro-Ins + insulin + E2 induced the expression of GLUT4 protein (p = 0.045 and p = 0.035, respectively) (**Figure 7B**). Additionally, a robust expression of p-GLUT4 was displayed in mature adipocytes when cells were treated with all treatments except D-Chiro-Ins alone (E2: p = 0.027; INS: p = 0.0023; E2 + INS: p = 0.003; D-Chiro-Ins + E2: p = 0.019; D-Chiro-Ins + INS: p = 0.028; D-Chiro-Ins + E2 + INS: p = 0.003) (**Figure 7C**). As expected, the p-GLUT4/GLUT4 ratio was found to be increased in presence of E2, insulin and its combination (p = 0.016, p = 0.01 and p = 0.031, respectively) (**Figure 7D**). These experiments suggest that D-Chiro-Ins, under insulin-stimulated conditions, regulates GLUT4 expression in human adipocytes.

## Discussion

This study gives clearer insights on the direct regulation exerted by D-Chiro-Inositol (D-Chiro-Ins) on human adipocytes differentiation, as well as its synergistic signaling activity with estrogen and with insulin. These results reinforce the hypothesis that the different D-Chiro-Ins clinical actions may depend on multiple targets, including the direct modulation of fat insulin signaling.

D-Chiro-Ins is a known regulator of insulin secretion in pancreatic β-cells where it also acts as a second messenger in the insulin receptor signaling pathway. D-Chiro-Ins also modulates the storage of sugar as glycogen and the respiratory chain for the production of ATP in rat adipocytes (40, 41). Our manuscript shows that similar regulatory actions of D-Chiro-Ins are also exerted in human adipocytes.

D-Chiro-Ins does not modify adipocytes viability or proliferation, while it regulates the differentiation of preadipocytes into mature adipocytes. D-Chiro-Ins acts on the accumulation of lipids and on the number and size of lipid droplets in the later stages of adipocyte differentiation, when SGBS cells reach a white adipose tissue phenotype, with preadipocytes and brown/beige adipocytes not being affected by D-Chiro-Ins.

The reasons for this selectivity of action on mature cells have to be explored but may be linked to the progressive increase in expression of inositol transporters during the adipocyte differentiation process, but this hypothesis has to be explored with specific studies. Estradiol also enhanced adipocyte differentiation and lipid storage at an earlier stage of differentiation since at this stage preadipocytes express only ERα (42). Later, estrogens control lipogenesis in mature adipocytes through the expression of both fat estrogen receptors: ERα and ERβ (13). Some possible mechanistic explanations for these findings have been published. Dos Santos et al. showed that 17β-estradiol (E2) inhibits apoptosis in human preadipocytes thanks to a crosstalk between the insulin growth factor-1 (IGF1) and ER-signaling pathways (43). Dieudonne et al. also showed that estrogens promote adipocyte differentiation in rats by interacting with IGF1 receptor and peroxisome proliferator-activated receptor-γ 2 (PAPP-γ 2) (44). The direct action of D-Chiro-Ins on human adipocytes leading to enhanced lipid storage and increased adipocyte size may in part explain why clinical use of D-Chiro-Ins has been associated to decreases in body mass index (22, 45). Indeed, effective adipocyte lipid storage alleviates insulin resistance (IR) and decreases triglycerides accumulations in muscles, liver and pancreatic islets (46). In line with this hypothesis, studies in rodents showed that myo-inositol strongly inhibits basal lipolysis in mature adipose cells and decreases Not Esterified Fatty Acids levels in plasma (47, 48). Hormone-insensitive basal lipolysis has been observed in enlarged mature adipocytes, and elevated levels of circulating free fatty acids decrease glucose utilization in muscle cells and stimulate hepatic glucose production. Thus, to increase insulin sensitivity, it is critical to prevent the production of excessive free fatty acids (47). So, the production of bigger lipid droplets and the higher lipid storage induced by D-Chiro-Ins may be involved in the decreased release of free fatty acids observed in animals and humans exposed to dietary supplementation with D-Chiro-Ins. These actions may be added to recent observation that D-Chiro-Ins exerted a stable hypoglycemic effect in mice, by means of enhancing hepatic glycogen storage (49, 50).

The analysis of the effects of D-Chiro-Ins on the expression and function of transducers of the insulin receptor in mature adipocytes provides additional insights. Our results show that D-Chiro-Ins alone and in combination with insulin and estradiol increased expression and promoted activation of IRS1, which is the main downstream effector of insulin receptor, these effects turned into an increased pIRS1/IRS1 ratio. D-Chiro-Ins did not increase the expression of Akt nor its phosphorylation/activation, suggesting that D-chiro-Ins effect may not selectively involve Akt pathway; this finding is in line with previous studies that have shown that some D-Chiro-Ins based compounds can activate alternative pathway as phosphoinositide-3 kinase (PI3K) (41). Additionally, we found that D-Chiro-Ins in combination with insulin and E2 turned into an increased expression and promoted activation of GLUT4. These results are in line with rat studies were oral administration of D-Chiro-Ins increased plasma membrane GLUT4 content in muscle cells (50). This confirms that D-Chiro-Ins is involved in the signal transduction pathway of insulin receptor and this action is evident also in adipocytes. This is also in line with the pioneering hypothesis by Joseph Larner et al., that defective D-Chiro-Ins may be a major contributor to insulin resistance, since D-Chiro-Ins turns out to act as a direct cofactor in the molecular signaling of IR (24).

Overall, these results suggest that E2 and D-Chiro-Ins can interact with insulin receptor signal transduction at different levels. E2 acting through a parallel, ER-mediated, recruitment of PI3K, and D-Chiro-Ins by working as an adapter/facilitator of insulin receptor (14). Such results clearly might help in a better understanding in regards of the metabolic changes and the increase of IR that occur in peri and menopausal women (51). Indeed, the physiological IR that typically occurs with the growing evidence of the estrogenic decay might be, at least in part, explained by the loss of synergy between estradiol and D-Chiro-Ins on fat tissue. Moreover, the relevant role exerted by D-Chiro-Ins on glucose and lipid metabolisms, that we disclosed in the present study, may explain, at least in part, the defective metabolic control observed in diabetic subjects, in PCOS and/or in PCOS patients with first grade relatives that is related to a reduced epimerase expression/function (26, 52, 53).

Our work has several limitations. In this manuscript we did not analyze the molecular pathways of adipocyte differentiation that are influenced by D-Chiro-Ins nor the potential implication of other insulin-related proteins that may be relevant to the synergistic effects on adipocyte function of D-Chiro-Ins, estrogen and insulin. To support the present results additional effects of D-Chiro-Ins on adipocyte function need to be characterized, such as glycogen formation and the related regulatory mechanistic steps, which will be the subject of a subsequent study. It is also relevant to notice that all the experiments were performed with physiological concentrations of glucose and insulin. It needs therefore to be assessed whether D-Chiro-Ins affects adipocyte function in the same way in culture conditions mimicking IR or diabetes. For instance, there is little information available to support whether a defective epimerase may lead to lower D-Chiro-Ins conversion and therefore to an insulin resistant state (20). In parallel, it is not clear if D-Chiro-Ins acts more as an insulin mimetic agent in insulin resistant adipocytes. The site of production of D-Chiro-Ins is not ascertained, as well. Indeed, D-Chiro-Ins levels in rodents seem to be derived from dietary sources as opposed to endogenous production (54).

On the same register, it will be of interest to characterize if concentrations of estradiol typical of pregnancy or post-menopause would still synergize with D-Chiro-Ins in enhancing insulin receptor signaling in adipose cells. The concentration of D-Chiro-Ins that we used for the *in vitro* study may also be a matter of debate. The concentration was chosen on the basis of previous studies on other tissues and based on viability tests. In clinical use, the usual therapeutic dose is 1000 mg per day, but lower or higher amounts are used. No information is available to estimate the concentration of active molecule that reaches adipose tissue during clinical use, thus evidence guiding correct dose selection for *in vitro* studies is missing.

In conclusion, D-Chiro-Ins is an intriguing molecule with many potential applications in clinical conditions where impaired insulin metabolism is encountered (55). D-Chiro-Ins is frequently used as a supplement in presence of IR but a lot needs to be done to fully characterize its pharmacokinetics and pharmacodynamics, and ultimately the biochemical and molecular mechanisms that explain its metabolic effects. This study discloses the actions of inositols on human fat cells demonstrating that D-Chiro-Ins can regulate directly human adipocytes, by enhancing their differentiation and insulin receptor signaling in synergy with estrogen. These observations lay the basis for a better understanding of the actions of D-Chiro-Ins in conditions were defective insulin signaling impairs metabolism in humans. Obviously, further research is needed to better assess whether adipose tissue can be pharmacologically targeted with D-Chiro-Ins to improve metabolic performance during pregnancy or menopause, or in women with PCOS.

## Data Availability Statement

The original contributions presented in the study are included in the article/supplementary material. Further inquiries can be directed to the corresponding author.

## Author Contributions 

MMG and TS conceived the study design, performed data interpretation and manuscript preparation. MMG, MF, TF, and IM performed the molecular studies. AF and AG for assistance in the manuscript preparation. All authors contributed to the article and approved the submitted version.

## Funding

This work was supported by an unrestricted grant from Farmitalia, the manufacturer of D-Chiro-inositol, (grant number: 539901_2021) and by the University of Pisa (grant number: 539999_2016), both funds to Tommaso Simoncini, Pisa, Italy. The funders had no role in the study design, data collection and analysis, in neither the decision to publish, nor preparation of the manuscript.

## Conflict of Interest

The authors declare that the research was conducted in the absence of any commercial or financial relationships that could be construed as a potential conflict of interest.
